# Effects of stable and fluctuating soil water on the agronomic and biological performance of root vegetables

**DOI:** 10.3389/fpls.2024.1325078

**Published:** 2024-02-14

**Authors:** Ge Li, Guolong Zhu, Jian Liu, Zhuan Wang, Huaiyu Long, Renlian Zhang, Kefan Yu

**Affiliations:** ^1^ State Key Laboratory of Efficient Utilization of Arid and Semi-arid Arable Land in Northern China/Institute of Agricultural Resources and Regional Planning, Chinese Academy of Agricultural Sciences, Beijing, China; ^2^ Institute of Farmland Irrigation, Chinese Academy of Agricultural Sciences, Xinxiang, Henan, China; ^3^ Beijing Liangxiang Lanxin Hydraulic Engineering & Design Co., Ltd, Beijing, China; ^4^ Department of Soil and Land Use, Norwegian Institute of Bioeconomy Research (NIBIO), Viken, Norway

**Keywords:** cherry radish, fluctuating soil water, negative pressure irrigation, stable soil water, water use efficiency

## Abstract

Compared to fluctuating soil water (FW) conditions, stable soil water (SW) can increase plant water use efficiency (WUE) and improve crop growth and aboveground yield. It is unknown, however, how stable and fluctuating soil water affect root vegetables. Here, the effects of SW and FW were studied on cherry radish in a pot experiment, using negative pressure irrigation and conventional irrigation, respectively. The assessed effects included agronomic parameters, physiological indices, yield, quality and WUE of cherry radish. Results showed that under similarly average soil water contents, compared with FW, SW increased plant photosynthetic rate, stomatal conductance and transpiration rate, decreased leaf proline content by 13.7–73.3% and malondialdehyde content by 12.5–40.0%, and increased soluble sugars content by 6.3–22.1%. Cherry radish had greater biomass accumulation and nutrient uptake in SW than in FW. Indeed, SW increased radish output by 34.6–94.1% with no influence on root/shoot ratio or root quality. In conclusion, soil water stability affected directly the water physiological indicators of cherry radish and indirectly its agronomic attributes and nutrient uptake, which in turn influenced the crop biomass and yield, as well as WUE. This study provides a new perspective for improving agronomy of root crops and WUE through managing soil water stability.

## 1 Introduction

Soil water is crucial for plant growth and development (Baier, 1965; Hey et al., 2020), and eventually affects agricultural productivity and sustainability (Leghari et al., 2021). In many growing environments ranging from arid to semi-humid, a moderate increase in soil water content (SWC) by irrigation can boost crop yield, and an optimal SWC can maximize the water use efficiency (WUE) of plants (Jahromi et al., 2023). Previous studies on soil water–plant relations have mostly focused on the SWC, however, there has been relatively limited understanding on the effects of soil water stability, i.e., stable soil water (SW) versus fluctuating soil water (FW), on crop growth and productivity (Niu et al., 2022).

In recent years, a pressure potential difference-crop initiate drawing water technique, namely as negative pressure irrigation (NPI) technology has been developed and proved as an effective irrigation method for water supply, with many potential advantages over traditional irrigation (Yang et al., 2022a). This technology uses a water potential difference caused by the negative pressure system, soil evaporation and plant transpiration to control water supply, and it can continuously and stably supply water to plants, which helps manage soil water fluctuations (Yang et al., 2022a). The technology can not only be used for studying SWC effects on crop performance, but also for assessing the effects of soil water stability (Zhang et al., 2021b; Niu et al., 2022).

Previous research on soil water stability is limited and has focused mainly on cash crops with aboveground yield. For example, Wang (2020) found that high-stability soil moisture boosted plant development and nutrient uptake of romaine lettuces (*Lactuca sativa* L.), with increased photosynthesis and WUE. In another study on lettuce, Gao et al. (2021) discovered that maintaining a stable water states considerably improved lettuce yield and quality, enhanced water utilization and decreased soil nitrogen loss as compared to fluctuating water conditions. On tomatoes (*Solanum lycopersicum* L.) and cucumbers (*Cucumis sativus* L.), Li et al. (2021b) confirmed that SW increased crop water productivity via maintaining soil water and nitrogen homogeneity. Elsewhere, long-term and stable irrigation during the tobacco (*Nicotiana tabacum* L.) growing period was found to minimize water consumption while increasing WUE, and the development and quality of flue-cured tobacco could be promoted by using appropriate SWC (Yang et al., 2022b). Similarly, studies on maize (*Zea mays* L.) have shown that SW could significantly promote economic factors in addition to growth and yield, compared with soil water state under wet and dry conditions (Zhang et al., 2021b; Zhang et al., 2022b). Despite these promising effects of SW conditions on the aboveground yield of the assessed crops (Niu et al., 2022; Yang et al., 2022b; Zhang et al., 2022a), their effects on root crops, particularly root vegetables, remain unknown.

Cherry radish (*Raphanus sativus* L. var. *radculus pers*) is a major root vegetable crop with highly nutritious and cherry-shaped root, a rapid growth cycle, and a short maturity period, as well as a substantial commercial value (Guo et al., 2019; Zhou et al., 2022). The growth and yield formation of radish were found to highly relate to the soil water conditions, for which proper water management increased radish yield and reduced radish root breaking (Kang and Wan, 2005; Lacerda et al., 2022). Optimizing the soil water management and planting date was found to boost radish production while reducing nitrogen loss by 40–50% (Zhang et al., 2021a), however, inappropriate water treatment was reported to have a detrimental impact on the growth and development of radish, resulting in inferior quality and yield penalties (Zomerfeld et al., 2021). Shafiq et al. (2015) found that water deficiency dramatically decreased the fresh weight of radish roots, increased the accumulation of carotenoids, malondialdehyde, glycine betaine and total soluble protein, and enhanced the antioxidant enzymes activity in radish roots. Another study has discovered that the temporal and spatial distribution of soil water, the distribution of radish root and the quality of the fruit were all strongly affected by soil water potential (Kang and Wan, 2005). Radish root development increased when the soil water potential decreased from –15 kPa to –55 kPa, although evapotranspiration decreased (Kang and Wan, 2005). Moreover, over-saturated soil decreased the rate of carbon assimilation by radish, hence limiting their shoot and root growth (Henschel et al., 2022).

Nevertheless, most of the studies focus on SWC wherein there is not much focus on soil water stability. Hence, the present study throws much insights on radish-soil water stability relations with the objectives: 1) to examine the effects of stable and fluctuating soil water on cherry radish growth and development, physiological indices, yield, quality, nutrient uptake, and WUE; 2) and to find out the optimal soil water conditions for root crops. We hypothesized: soil water stability could affect root crops morphological characteristics and physiological responses, and compared to FW, SW could sustain or improve radish growth, biomass accumulation and yield. It was expected to lay the foundation for revealing the relationship between soil water stability and root vegetables.

## 2 Materials and methods

### 2.1 Experimental site and climatic condition

The experiment was conducted in a rainproof shelter located at the Chinese Academy of Agricultural Sciences in Beijing, China (39.6°N, 116.2°E) from August through October in 2019. The study region has a typical continental climate being characterized as warm-temperate and semi-humid, with hot and rainy summers and cold and dry winters. The annual mean temperature was 10–12°C, and the annual frost-free period was 180–220 days. In this experiment, cherry radish was used as an experimental root vegetable, and it was grown in pots with dimensions of 42 cm in length, 26 cm in width, and 25 cm in height. The experimental soil particle distribution and chemical properties were listed in **Table 1**.

**Table 1:** Soil particle distribution and physicochemical properties.

Particulars	Values
Mechanical analysis	
Clay (%)	24.5
Silt (%)	22.5
Sand (%)	52.9
Textural class	Clay loam
Physical Properties	
Bulk density (g cm^–3^)	1.4
Chemical properties	
Soil pH (1:5 soil: water ratio)	6.66
Total nitrogen (g kg^–1^)	1.35
Total phosphorus (g kg^–1^)	0.59
Alkali hydrolyzed nitrogen (mg kg^–1^)	93.5
Available phosphorus (mg kg^–1^)	34.3
Available potassium (mg kg^–1^)	194
Organic matter (g kg^–1^)	16.0

### 2.2 Negative pressure irrigation device

Soil water was studied under water supply from NPI devices that were developed by the Chinese Academy of Agricultural Sciences (Chinese Patents. ZL201110093923.2 and ZL201310554433.7). A NPI device (described in detail in Niu et al., 2022; Yang et al., 2022a) is consisted of a negative pressure controller, a water supply bucket (13.1 cm inner radius) and an irrigator (porous ceramic pipe, 26 cm long, 19 mm outer diameter and 10 mm internal diameter). Under the NPI system, there is a dynamic balance between soil matrix potential and irrigation pressure, thereby maintaining the stable water supply and a SW condition.

### 2.3 Experimental design and crop management

Six water treatments, each with six replicates, were established: NPI at –2 kPa (NPI0), –5 kPa (NPI1) and –8 kPa (NPI2) based on previous experimental results (Zhu et al., 2022), and conventional flood irrigation (CI) at 80%–90% field capacity (FC) (CI1), 70%–90% FC (CI2) and 60%–90% FC (CI3), respectively. The daily irrigation amount for NPI treatments was calculated by multiplying the water level difference by the cross-sectional area of the water supply bucket every day, and the cumulative irrigation amount was obtained by adding the daily irrigation amounts. When the SWC approached or dropped below the lower limits of CI treatments, irrigation was triggered to reach the upper limits and the irrigation amount was calculated according to the measured SWC, the set upper irrigation limits, and the soil volume in the pot (Wang, 2020).

Cherry radish was sown on August 10, 2019, for which each pot was grided to six sowing sites and two seeds per site. Each pot included 22 kg of air-dried soil with a bulk density of 1.4 g cm^–3^ and a FC of 29.1% (v/v). Before sowing, the same fertilizer amounts (2.8 g urea, 5.2 g superphosphate, and 5.0 g potassium sulfate) were applied to each pot and all pots were irrigated to 100% FC. On August 22, 2019, the plants were thinned to six seedlings each pot. Water treatments were started on August 29, 2019 and completed when the radish was harvested on September 20, 2019.

### 2.4 Sampling and measurements

#### 2.4.1 Determination of SWC and soil water fluctuant parameters

A soil water velocity tester (AZS-100, Beijing Aozuo Ecological Instrument Co., Ltd, China) was used to measure the volumetric SWC every 2 days under NPI treatments and every day under CI treatments. The measurement was done between 17:00 and 18:00 at the center of the growth pot, where three points with an equal distance were chosen to determine SWC.

Equation (1) was used to calculate the coefficient of variation (*CV*) of soil water (Wang, 2020):


(1) CV=SD/θ

where *SD* is the standard deviation of SWC at different days, *θ* is the mean SWC. If *CV* ≤ 0.1, the soil water belongs to weak variation, if 0.1 < *CV* < 1, it belongs to medium variation, and if *CV* ≥ 1, it belongs to strong variation. The stronger the soil water fluctuation, the higher the value.

The fluctuation coefficient (*δ*) of soil water was computed using Equation (2):


(2) $$\delta =\frac{1}{n-1}\sum^{}\frac{2\left|\theta_{i}-\theta_{i-1}\right|}{\theta_{i}+\theta_{i-1}}$$

where *θ_i_* is the SWC on the i^th^ day, *θ_i_*_–_ *_1_* is the SWC on the (i–1)^th^ day, and *n* is the number of SWC observation days. The magnitude of *δ* reflects the soil water stability, and the smaller the value is, the more stable the soil water.

#### 2.4.2 Determination of growth and leaf physiological indicators

Cherry radish plant height, maximum leaf length and maximum leaf width were measured on the 11^th^ and 21^th^ days after treatment (DAT). Plant height was measured from the ground surface to plant’s tip and a ruler was used to measure the leaf’s length and width of maximum leaf.

Gas exchange parameters, such as photosynthetic rate (Photo), stomatal conductance (Cond) and transpiration rate (Trmmol), were recorded using a portable photosynthesis system (Li-6400XT, LI-Cor, NE, USA) at 8:30–11:30 am on the 11^th^ and 21^th^ DAT. The photosynthetic active radiation was set to 1000 µmol m^−2^ s^−1^, the flow rate was set to 500 µmol s^−1^, and an open gas circuit was used. Leaf water use efficiency (WUE_L_) was calculated according to Sun et al. (2018), as shown in Equation (3):


(3) $$WUE_{L}=Photo/Trmmol$$

where *WUE_L_* is the leaf water use efficiency (μmol CO_2_ mmol^–1^ H_2_O), *Photo* is the photosynthetic rate (µmol CO_2_ m^–2^ s^–1^), and *Trmmol* is the transpiration rate (mmol H_2_O m^–2^ s^–1^).

For the purpose of estimating the physiological response of plants to water treatment, the contents of proline, malondialdehyde, and soluble sugars were measured at the National Energy R&D Center for Non-food Biomass, China Agricultural University, Beijing, China. The leaves of cherry radish were collected, immersed in liquid nitrogen, frozen for 30 min, and then stored in a freezer at –80°C. Thereafter the analyzes were done using high performance liquid chromatography method for free proline contents, a thiobarbituric acid method for malondialdehyde contents, and anthrone colorimetry method for soluble sugars contents as given by Zhang et al. (2022b).

#### 2.4.3 Determination of radish biomass, yield and quality

At harvest, the cherry radish was harvested and immediately weighed, and the diameter of the radish bulb was measured using a vernier caliper. The yield of cherry radish was considered as the fresh weight of radish bulb. Thereafter, they were dried in an oven at 105°C for 30 min before being dried at 75°C to a constant weight to determine the biomass (Li et al., 2021a; Guo et al., 2022).

Various quality parameters were determined on the radish root. The nitrate content was determined following the methodology described by GB5009.33-2016. The vitamin C content was determined using the high-performance liquid chromatography method, reducing sugar content was determined using the colorimetric method, and soluble protein content was determined using the Coomassie brilliant blue method (Ouyang et al., 2021; Zhang et al., 2022b).

#### 2.4.4 Determination of plant nutrient uptake

After measuring biomass, the aboveground (stems and leaves) and roots were ground, and a subsample was digested for the determination of plant nutrient content. Total nitrogen content was determined in plant samples using the Kjeldahl method, as described by Bao (2000). Total phosphorus and total potassium contents were determined using the inductively coupled plasma-optical emission spectrometry with microwave digestion (Wang, 2020). The uptake of nitrogen, phosphorus and potassium was calculated according to (Yang et al., 2020), as shown in Equation (4):


(4) Nutrient uptake=Nutrient content × DM

where *Nutrient uptake* is the uptake of nitrogen, phosphorus or potassium (mg plant^–1^), *Nutrient content* is the content of nitrogen, phosphorus or potassium (g kg^–1^), and *DM* is the dry matter mass (g plant^–1^).

#### 2.4.5 Evapotranspiration and WUE

The evapotranspiration of cherry radish during the experiment was calculated using the water balance method (Ertek et al., 2006; Qu et al., 2020), as expressed in Equation (5):


(5) $$ET=I–\Delta s=I–\left(\theta –\theta_{0}\right)\;\times \;V$$

where *ET* is the evapotranspiration (L), *I* is the irrigation amount (L), *Δs* is the change in soil water storage (L) between the beginning and the end of the experiment, *θ_0_* and *θ* refer to the observed SWC (%) at the beginning and the end of the experiment, respectively, and *V* is the soil volume in the pot (L).

Water use efficiency was calculated according to (Sun et al., 2018; Liao et al., 2022), as shown in Equations (6, 7):


(6) $$WUE_{Y}=Y/ET$$

(7) $$WUE_{B}=B/ET$$

where *WUE_Y_* is the yield water use efficiency (g kg^–1^), *Y* is the fresh yield of cherry radish bulbs (g plant^–1^), *ET* is the evapotranspiration (L plant^–1^), *WUE_B_* is the biomass water use efficiency (g kg^–1^), *B* is the biomass i.e. the dry matter mass of cherry radish, including both the above- and underground parts (g plant^–1^).

### 2.5 Statistical analysis

The data were processed with Microsoft Excel 2010 (Microsoft Crop, USA) and presented as mean ± standard deviation. SPSS 22.0 (SPSS, Chicago, USA) was used to perform analysis of variance (ANOVA) among the water management treatments, following the Duncan’s multiple-range test. Significant differences between treatments at the *P* < 0.05 level were indicated with different letters. The software Origin Pro 2021 (OriginLab Corporation, Northampton, MA, USA) was used for making graphs. Person’s correlation and principal component analysis (PCA) were used to evaluate the relationship between soil water and plant parameters of cherry radish. To further investigate the relationships between agronomic traits, physiological indices, yield and WUE, a partial least squares path model (PLS-PM) was constructed using the “plspm” package in R language (4.1.1) (Sanchez et al., 2015). The quality of the PLS-PM was evaluated by examining the goodness-of-fit index (GOF), in which a value > 0.7 indicates the acceptable overall prediction performance of the model (Chen et al., 2022).

## 3 Results

### 3.1 Soil water parameters

**Figure 1:**
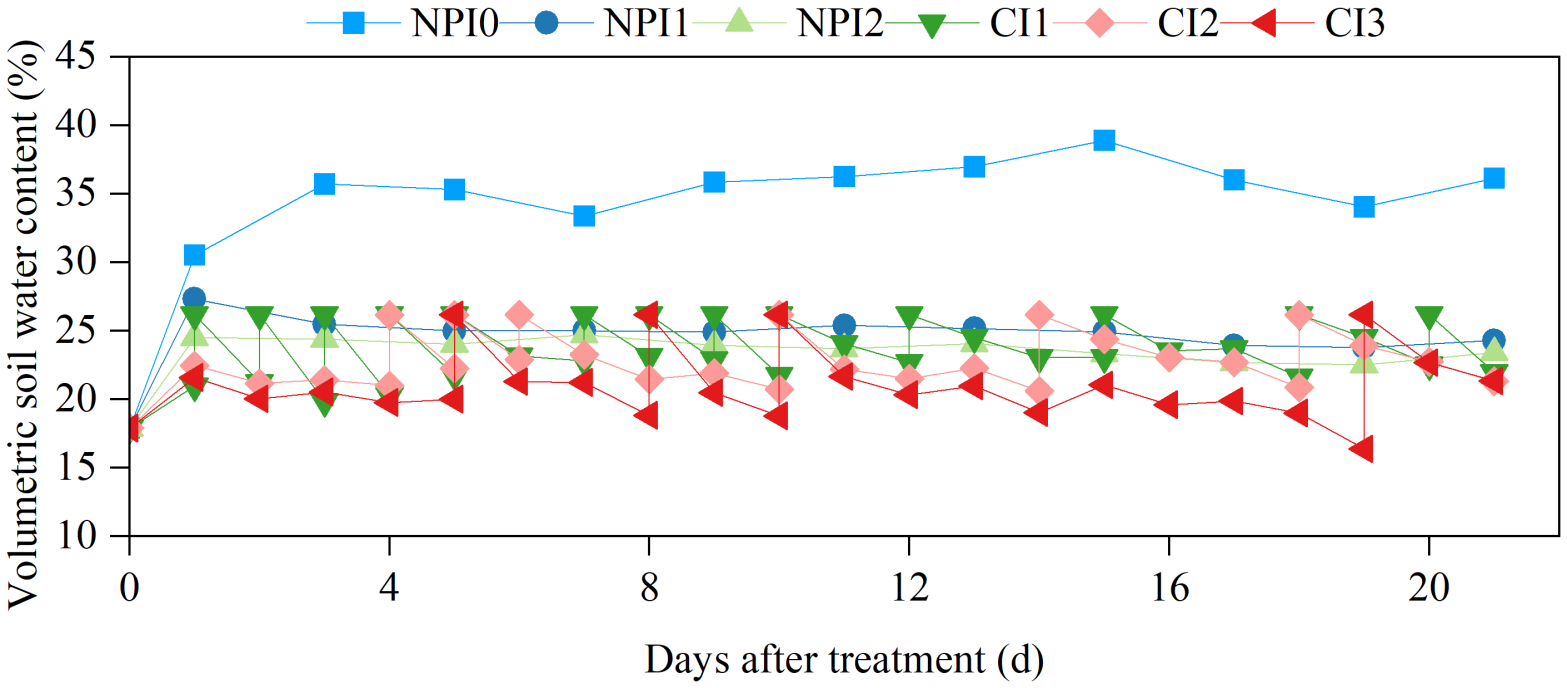
Temporal dynamics of volumetric soil water content under different treatments. NPI, negative pressure irrigation; CI, conventional irrigation.

The SWC under the NPI0, NPI1, and NPI2 treatments changed only slightly over time, and the coefficients of variation and fluctuation were relatively small, indicating a stable moisture status (**Table 2** and **Figure 1**). The SWC in the CI1, CI2, and CI3 treatments varied greatly as time progressed, exhibiting a “sawtooth” shape, and the soil water fluctuant parameters were significant, indicating fluctuating moisture conditions. Mean SWC values were similar between NPI1 (86% FC) and CI1 (85% FC) treatments and between NPI2 (82% FC) and CI2 (80% FC) treatments, which suggested that the SW and FW comparison was well established with similar overall SWC conditions.

**Table 2:** Soil water content (SWC) and soil water fluctuant parameters under different treatments.

Treatment	Range of SWC (%)	Mean SWC (%)	Variation coefficient	Fluctuation coefficient
NPI0	33.8–38.9 (116%–134% FC)	35.4 (122% FC)	0.06	0.06
NPI1	23.8–25.5 (82%–88% FC)	25.0 (86% FC)	0.04	0.02
NPI2	22.5–24.7 (77%–85% FC)	23.8 (82% FC)	0.03	0.02
CI1	23.3–26.2 (80%–90% FC)	24.7 (85% FC)	0.10	0.14
CI2	20.4–26.2 (70%–90% FC)	23.3 (80% FC)	0.10	0.10
CI3	17.4–26.2 (60%–90% FC)	21.8 (75% FC)	0.12	0.13

NPI, negative pressure irrigation; CI, conventional irrigation. FC, field capacity.

### 3.2 Variation of agronomic traits

**Figure 2:**
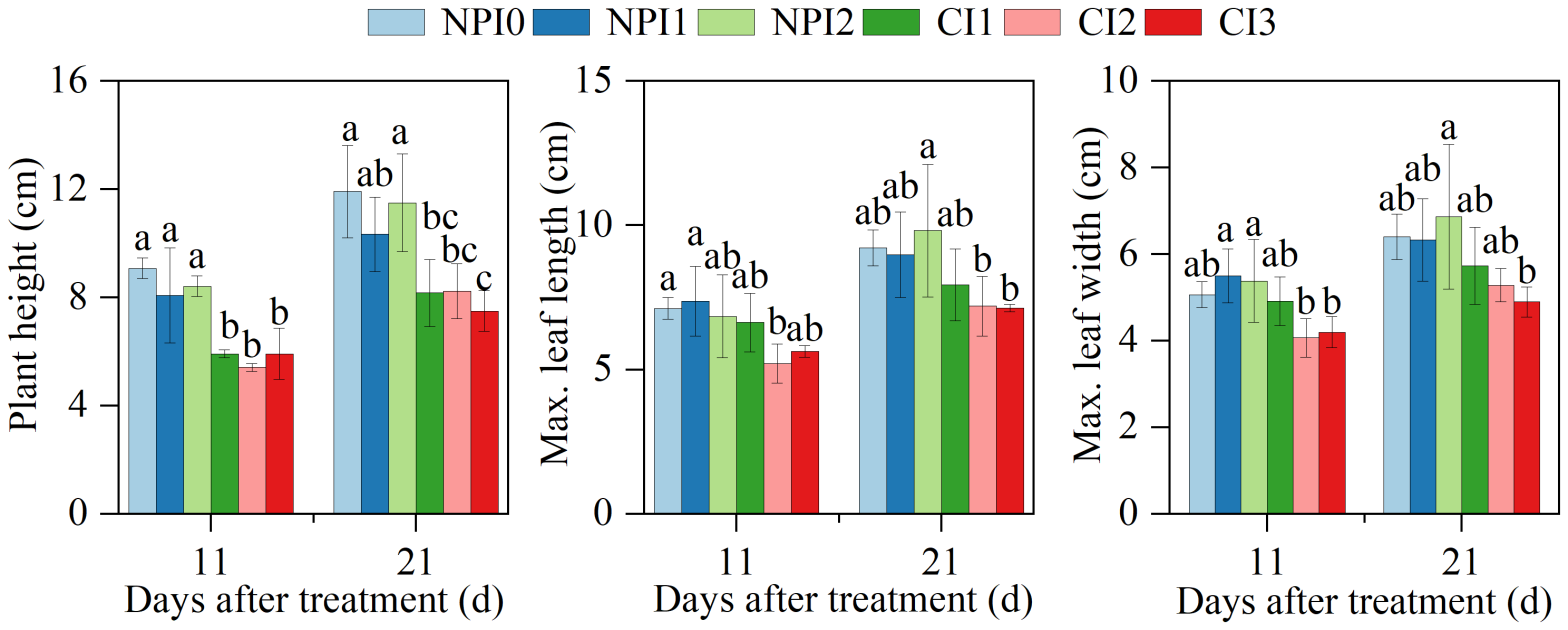
Variations of plant height, leaf length and leaf width under different treatments during cherry radish growing seasons. NPI, negative pressure irrigation; CI, conventional irrigation. Different lowercase letters above the columns indicate significant differences among treatments at the *P* < 0.05 level within the same day.

Agronomic traits of plants dynamically reflect their growth and development processes, which are closely linked to crop yield (Liu et al., 2019; He et al., 2020). The NPI treatments increased the radish plant height, maximum leaf length and maximum leaf width as compared to CI under similar SWC conditions (**Figure 2**). Specifically, NPI1 treatment raised plant height by 26.5%, maximum leaf length by 13.2%, and maximum leaf width by 10.5% on 21 DAT, compared to CI1 treatment; NPI2 treatment increased plant height by 39.7%, maximum leaf length by 36.3%, and maximum leaf width by 30.0%, compared to CI2 treatment. There was no significant difference in agronomic traits between SWC treatments under NPI or CI conditions. The results showed that under similar SWC conditions SW improved the growth and development of cherry radish compared to FW.

### 3.3 Leaf photosynthetic parameters

**Figure 3:**
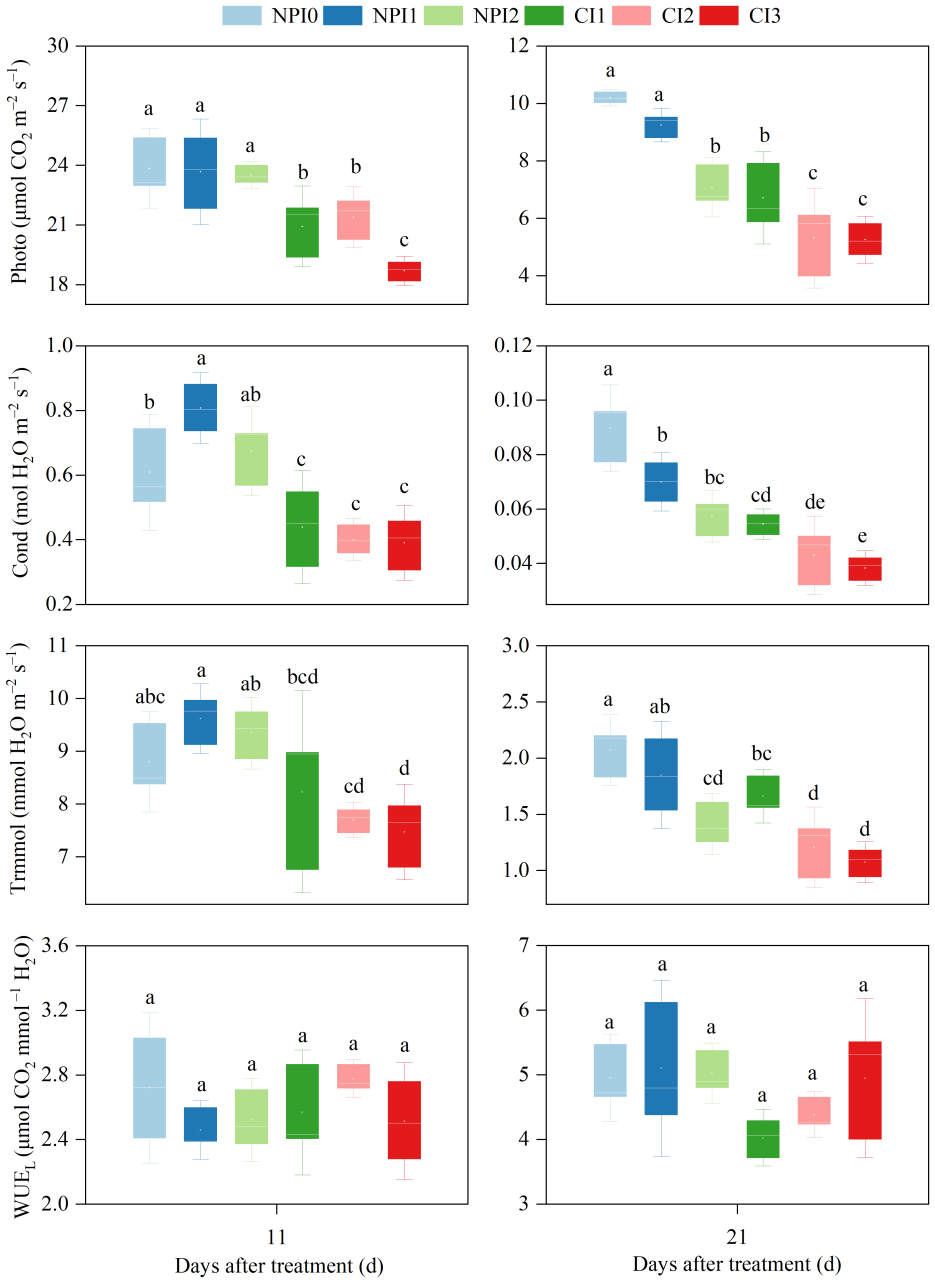
Variations of gas exchange parameters and WUE_L_ under different treatments during cherry radish growing seasons. NPI, negative pressure irrigation; CI, conventional irrigation; Photo, photosynthetic rate; Cond, stomatal conductance; Trmmol, transpiration rate; WUE_L_, leaf level water use efficiency. Different lowercase letters above the boxes indicate significant differences among treatments at the *P* < 0.05 level within the same day.

Photosynthesis is the fundamental physiological process for producing plant material. Gas exchange characteristics, including Photo, Cond, and Trmmol were affected by different water scheduling. The Photo of plants under NPI treatments on 21 DAT increased by 44.0% when SWC rose from 82% FC to 122% FC, and the leaf Photo under CI treatments increased by 27.8% when SWC rose from 75% FC to 85% FC (**Figure 3**). In 11–21 DAT, NPI1 treatment increased the Photo of cherry radish by 13.1–37.7%, Cond by 28.4–84.0%, and Trmmol by 11.3–16.9%, compared with CI1 treatment; NPI2 treatment could enhance the Photo by 9.9–33.4%, Cond by 33.2–68.3%, and Trmmol by 17.1–21.4%, compared to CI2 treatment. The results exhibited that SW was more favorable to improving the Photo, Cond, and Trmmol of cherry radish than FW under similar SWC circumstances; however, there was no discernible difference between SW and FW in terms of WUE_L_.

### 3.4 Leaf proline, malondialdehyde and soluble sugars

Plants under water stress accumulate free proline and soluble sugars to regulate their osmotic potential, thus improving their growth characteristics and tolerance to water deficit (Silva and Arrabaça, 2004; Yooyongwech et al., 2016). An increase in the malondialdehyde content is a common response of stressed plants, water stress could lead to the overproduction of malondialdehyde, which causes cell membrane damage and, ultimately, plant cell death (Abbasi et al., 2020). The contents of free proline and malondialdehyde in leaves treated with NPI treatments were generally lower than those treated with CI treatments, while the content of soluble sugars was greater than that of CI treatments (**Table 3**). When SWC was approximately 85% FC, NPI treatments decreased the free proline contents by 13.7% and malondialdehyde contents by 12.5%, while increasing the soluble sugars content by 22.1% compared to CI treatments. When SWC was approximately 80% FC, compared to CI treatments, NPI treatments decreased free proline contents by 73.3% and malondialdehyde contents by 40.0%, but increased soluble sugars content by 6.29%. The CI2 treatment showed the highest free proline and malondialdehyde contents compared to other treatments. These results indicated that the water physiological indices of cherry radish leaves were moderately influenced by the soil water stability, with cherry radish leaves grown under SW having lower free proline and malondialdehyde contents and higher soluble sugars content than those grown under FW when SWC was essentially the same.

**Table 3:** Effects of different treatments on free proline, malondialdehyde and soluble sugars contents in cherry radish leaves.

Treatment	Free proline content (µg g^–1^)	Malondialdehyde content (µg g^–1^)	Soluble sugars content (g kg^–1^)
NPI0	11.1 ± 1.72b	0.72 ± 0.04b	37.8 ± 3.76a
NPI1	17.8 ± 0.99b	0.75 ± 0.22ab	35.1 ± 1.88ab
NPI2	18.9 ± 10.4b	0.82 ± 0.33ab	35.4 ± 2.51ab
CI1	20.6 ± 13.5b	0.86 ± 0.32ab	28.8 ± 1.71c
CI2	70.8 ± 5.00a	1.37 ± 0.52a	33.3 ± 1.31b
CI3	49.5 ± 34.1a	0.79 ± 0.30ab	27.6 ± 0.58c

NPI, negative pressure irrigation; CI, conventional irrigation. Different lowercase letters in the same column indicate significant differences among treatments at the P < 0.05 level.

### 3.5 Yield, biomass and radish quality

The yield and root transverse diameter of cherry radish in the NPI treatments declined as SWC fell, but there was no significant difference between treatments (**Table 4**). The yield, root transverse diameter, root longitudinal diameter, biomass, and root/shoot ratio of cherry radish under CI treatments reduced with decreasing SWC, but only 85% FC improved yield and root transverse diameter significantly compared to 80% FC and 75% FC. Comparing NPI1 treatment to CI1 treatment, there was a significant increase in cherry radish yield (34.6%), root longitudinal diameter (18.1%), and biomass (42.2%). Comparing NPI2 treatment to CI2 treatment, there was a substantial increase in yield significantly of 94.1%, root transverse diameter of 27.1%, root longitudinal diameter of 27.6% and biomass of 63.5%. SW was conducive to the accumulation of biomass, whereas soil water stability had no significant effect on the root/shoot ratio.

**Table 4:** Yield, root morphology, biomass and root/shoot ratio of cherry radish under different treatments.

Treatment	Yield (g plant^–1^)	Root transverse diameter (mm)	Root longitudinal diameter (cm)	Biomass (g plant^–1^)	Root/shoot ratio
NPI0	16.5 ± 3.10a	26.4 ± 2.89a	4.31 ± 0.26a	1.83 ± 0.02a	0.85 ± 0.17a
NPI1	16.0 ± 1.51a	26.1 ± 1.27a	3.99 ± 0.38a	1.70 ± 0.28a	1.05 ± 0.17a
NPI2	13.7 ± 2.19ab	23.8 ± 0.46a	4.01 ± 0.31a	1.71 ± 0.27a	0.84 ± 0.11a
CI1	11.9 ± 0.81b	24.2 ± 1.11a	3.38 ± 0.20b	1.19 ± 0.31b	1.02 ± 0.16a
CI2	7.04 ± 0.36c	18.7 ± 1.02b	3.14 ± 0.17b	1.05 ± 0.04b	0.94 ± 0.12a
CI3	5.54 ± 0.58c	17.7 ± 1.02b	2.96 ± 0.22b	0.87 ± 0.10b	0.85 ± 0.09a

NPI, negative pressure irrigation; CI, conventional irrigation. Different lowercase letters in the same column indicate significant differences among treatments at the P < 0.05 level.

Nitrate, vitamin C, reducing sugar, and soluble protein contents are quality parameters of radish (Yousaf et al., 2021; Huang et al., 2023). As shown in **Table 5**, there was no significant difference between NPI1 and CI1 treatments in terms of radish quality. NPI2 treatment significantly reduced soluble protein content by 28.6%, compared to CI2 treatment, but there were no significant differences in contents of nitrate, vitamin C, or reducing sugar. The highest values of nitrate, vitamin C and reducing sugar were observed in the CI2 treatment.

**Table 5:** Quality of cherry radish under different treatments.

Treatment	Nitrate content (g kg^–1^)	Vitamin C content (mg 100 g^–1^)	Reducing sugar content (g 100 g^–1^)	Soluble protein content (mg g^–1^)
NPI0	9.51 ± 0.88a	29.2 ± 10.7a	1.02 ± 0.19ab	0.38 ± 0.05ab
NPI1	7.85 ± 1.20a	26.0 ± 7.35a	0.51 ± 0.40b	0.36 ± 0.05abc
NPI2	7.72 ± 1.82a	20.6 ± 11.4a	1.09 ± 0.74ab	0.27 ± 0.01c
CI1	8.75 ± 2.46a	24.9 ± 7.97a	1.16 ± 0.32ab	0.29 ± 0.03bc
CI2	11.0 ± 2.15a	33.1 ± 10.4a	1.80 ± 0.30a	0.37 ± 0.08ab
CI3	8.21 ± 2.61a	25.6 ± 1.79a	1.05 ± 0.28ab	0.42 ± 0.05a

NPI, negative pressure irrigation; CI, conventional irrigation. Different lowercase letters in the same column indicate significant differences among treatments at the P < 0.05 level.

### 3.6 Content and uptake of nutrient

In the crop growth environment, the release, transformation, movement, and absorption of soil nutrients by plants were inseparable from soil water, and optimal soil water conditions were conducive to the uptake and utilization of nutrients by crops (Qu et al., 2020; Gao et al., 2021). Under similar SWC conditions, the nitrogen content of radish in the NPI treatments were significantly lower than those in the CI treatments. When SWC was about 85% FC, NPI treatments decreased nitrogen content by 10.8–11.0%, phosphorus content by 0.0–4.33% and potassium content by 10.5–20.3% compared with CI treatments. When SWC was close to 80% FC, in comparison to CI treatments, NPI treatments increased phosphorus content by 4.94–16.0%, but decreased nitrogen content by 6.32–18.2%.

In comparison to CI treatments, the uptake of total nitrogen, phosphorus, and potassium by cherry radish under NPI treatments increased by 50.8%, 72.2%, and 48.8%, respectively (**Table 6**). When SWC was about 85% FC, NPI treatments increased total nitrogen uptake by 25.9%, total phosphorus uptake by 40.3% and total potassium uptake by 24.9% compared with CI treatments; specifically, NPI increased nitrogen uptake by 23.6%, phosphorus uptake by 32.8%, and potassium uptake by 6.52% in stems and leaves; increased nitrogen uptake by 29.7%, phosphorus uptake by 46.7%, and potassium uptake by 29.9% in roots. When SWC was close to 80% FC, in comparison to CI treatments, NPI treatments increased total nitrogen uptake by 47.6%, total phosphorus uptake by 78.6%, and total potassium uptake by 48.2%; specifically, NPI increased nitrogen uptake by 60.9%, phosphorus uptake by 80.1% and potassium uptake by 26.1% in stems and leaves; increased nitrogen uptake by 25.7%, phosphorus uptake by 77.3%, and potassium uptake by 55.7% in roots. These results showed that SW enhanced the nutrients uptake of cherry radish compared to FW.

**Table 6:** Nutrient content and uptake of cherry radish under different treatments.

Item		NPI0	NPI1	NPI2	CI1	CI2	CI3
N content (g kg^–1^)	Stems & leaves	51.3 ± 1.97c	50.7 ± 0.61c	54.1 ± 1.36bc	57.0 ± 1.68ab	57.7 ± 3.56a	56.9 ± 0.41ab
	Roots	32.8 ± 1.82bc	31.6 ± 2.11c	30.4 ± 1.06c	35.4 ± 0.86ab	37.2 ± 1.49a	38.6 ± 2.41a
P content (g kg^–1^)	Stems & leaves	2.62 ± 0.20b	2.72 ± 0.07ab	2.90 ± 0.14a	2.85 ± 0.03ab	2.77 ± 0.14ab	2.69 ± 0.10ab
	Roots	3.18 ± 0.26b	3.43 ± 0.13ab	3.83 ± 0.37a	3.43 ± 0.32ab	3.30 ± 0.05ab	3.16 ± 0.40b
K content (g kg^–1^)	Stems & leaves	12.9 ± 1.81c	12.5 ± 0.67c	13.7 ± 0.86c	15.6 ± 3.49bc	18.7 ± 1.84ab	20.6 ± 2.01a
	Roots	55.8 ± 4.89a	54.7 ± 0.99a	60.0 ± 6.18a	61.1 ± 3.57a	58.9 ± 0.83a	57.7 ± 1.81a
N uptake (mg plant^–1^)	Stems & leaves	51.2 ± 5.51a	42.3 ± 9.64ab	50.1 ± 4.51a	34.2 ± 11.7bc	31.1 ± 1.20bc	26.6 ± 3.37c
	Roots	27.5 ± 4.09a	27.2 ± 3.42a	23.8 ± 4.43ab	21.0 ± 3.74abc	18.9 ± 2.36bc	15.3 ± 1.95c
	Total	78.7 ± 2.47a	69.5 ± 13.1ab	73.9 ± 8.41a	55.2 ± 15.5bc	50.1 ± 3.34c	42.0 ± 4.40c
P uptake (mg plant^–1^)	Stems & leaves	2.61 ± 0.23a	2.28 ± 0.53ab	2.69 ± 0.24a	1.71 ± 0.60bc	1.49 ± 0.01c	1.26 ± 0.12c
	Roots	2.68 ± 0.54a	2.95 ± 0.32a	2.98 ± 0.44a	2.01 ± 0.19b	1.68 ± 0.18bc	1.25 ± 0.06c
	Total	5.29 ± 0.42a	5.23 ± 0.84a	5.66 ± 0.56a	3.73 ± 0.78b	3.17 ± 0.16bc	2.50 ± 0.08c
K uptake (mg plant^–1^)	Stems & leaves	12.9 ± 2.70a	10.5 ± 2.99a	12.7 ± 0.77a	9.83 ± 5.41a	10.1 ± 0.74a	9.67 ± 1.53a
	Roots	46.7 ± 6.26a	47.0 ± 3.64a	46.6 ± 5.84a	36.2 ± 6.60b	29.9 ± 2.57bc	22.9 ± 2.26c
	Total	59.7 ± 4.81a	57.5 ± 6.62ab	59.3 ± 6.10a	46.0 ± 12.0bc	34.0 ± 1.95cd	32.6 ± 2.77d
Nutrient uptake ratio	N: P: K	1: 0.07: 0.76	1: 0.08: 0.83	1: 0.08: 0.80	1: 0.07: 0.83	1: 0.06: 0.80	1: 0.06: 0.78

NPI, negative pressure irrigation; CI, conventional irrigation; N, nitrogen; P, phosphorus; K, potassium. Different lowercase letters in the same column indicate significant differences among treatments at the P < 0.05 level.

### 3.7 Evapotranspiration and WUE

Under similar SWC conditions, radish WUE_Y_ and WUE_B_ in the NPI treatments were generally higher than those in the CI treatments (**Table 7**). Under NPI treatments, the evapotranspiration of cherry radish reduced as SWC decreased, the WUE_Y_ exhibited a tendency of initially increasing and then dropping with the decreasing SWC, and the WUE_B_ increased as SWC fell. Under CI treatments, evapotranspiration and WUE_Y_ decreased as SWC decreased, whereas WUE_B_ increased initially and then decreased as SWC decreased. The lowest value of evapotranspiration was observed in the CI3 treatment, while the NPI1 treatment showed the highest value of WUE_Y_ and the NPI2 treatment showed the highest value of WUE_B_ from all treatments. NPI1 treatment significantly improved WUE_Y_ by 45.9% and WUE_B_ by 53.8% compared to CI1 treatment, and NPI2 treatment remarkably increased WUE_Y_ by 74.2% and WUE_B_ by 46.7% compared to CI2 treatment.

**Table 7:** Effects of different treatments on evapotranspiration and water use efficiency of cherry radish.

Treatment	Evapotranspiration (L)	WUE_Y_ (g kg^–1^)	WUE_B_ (g kg^–1^)
NPI0	3.78 ± 0.67a	4.51 ± 1.38b	0.49 ± 0.08b
NPI1	2.29 ± 0.17bc	6.97 ± 0.25a	0.74 ± 0.07a
NPI2	2.08 ± 0.15bc	6.56 ± 0.68a	0.82 ± 0.07a
CI1	2.48 ± 0.03b	4.78 ± 0.27b	0.48 ± 0.12b
CI2	1.87 ± 0.04c	3.76 ± 0.24bc	0.56 ± 0.03b
CI3	1.85 ± 0.01c	2.99 ± 0.31c	0.47 ± 0.06b

NPI, negative pressure irrigation; CI, conventional irrigation; WUE_Y_, yield water use efficiency; WUE_B_, biomass water use efficiency. Different lowercase letters in the same column indicate significant differences among treatments at the P < 0.05 level.

### 3.8 Correlation analysis and PCA

**Figure 4:**
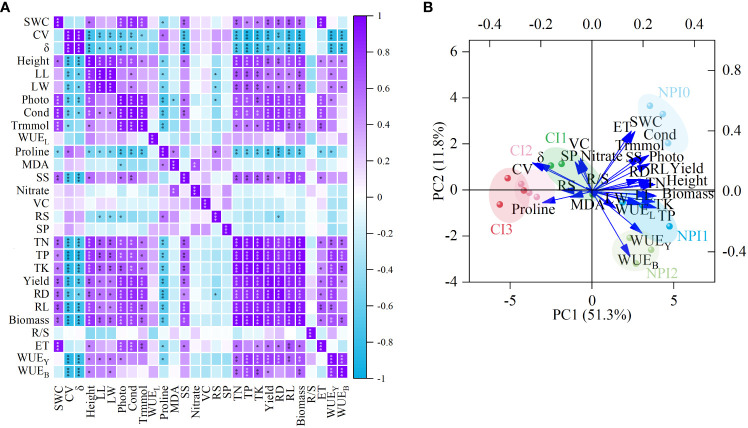
Correlation analysis **(A)** between soil water and plant indices, and biplot **(B)** of principal component analysis (PCA) of cherry radish indices under different treatments. The data used for agronomic traits and gas exchange parameters were the data at harvest time. SWC, soil water content; CV, variation coefficient; δ, fluctuation coefficient; Height, plant height; LL, leaf length; LW, leaf width; Photo, photosynthetic rate; Cond, stomatal conductance; Trmmol, transpiration rate; WUE_L_, leaf water use efficiency; Proline, free proline content; MDA, malondialdehyde content; SS, soluble sugars content; Nitrate, nitrate content; VC, vitamin C content; RS, reducing sugars content; SP, soluble protein content; TN, total nitrogen uptake; TP, total phosphorus uptake; TK, total potassium uptake; Yield, root yield; RD, root transverse diameter; RL, root longitudinal diameter; Biomass, total biomass; R/S, root/shoot ratio; ET, evapotranspiration; WUE_Y_, yield water use efficiency; WUE_B_, biomass water use efficiency; NPI, negative pressure irrigation; CI, conventional irrigation. *, ** and *** indicate significant differences at *P* < 0.05, *P* < 0.01 and *P* < 0.001 levels, respectively. PCn indicated the extracted principal component.

The data of plant indices response to soil water were presented in **Figure 4A**. The SWC was significantly positively correlated with gas exchange parameters, nutrient uptake, biomass, evapotranspiration, yield and root morphology of cherry radish, and significantly negatively correlated with the proline content. The soil water fluctuant parameters were significantly negatively correlated with the agronomic traits, Photo, Cond, leaf soluble sugars content, nutrient uptake, biomass, yield, root morphology, WUE_Y_ and WUE_B_ of cherry radish. Particularly, there was a significant negative correlation between photosynthetic parameters and the soil water fluctuant parameters (except for Trmmol and δ), and the correlation coefficient was between –0.50 and –0.65 (**Figure 4A**). Besides, the proline content of cherry radish leaves was significantly negatively correlated with SWC (r=–0.48) and positively correlated with the variation coefficient of soil water (r=0.57); the soluble sugars content of leaves had a significant positive correlation with SWC (r=0.59), and a significant negative correlation with the coefficients of variation and fluctuation of soil water (r=–0.72 and r=–0.76).

Principal component analysis exhibited clear distinctions between various water treatments (**Figure 4B**). Six principal components were extracted, of which PC1 (primarily correlated to CV, δ, Height, soluble sugars, nutrient uptake, biomass, yield, root longitudinal diameter, WUE_Y_ and WUE_B_) could explain 51.3% of the variables, PC2 (primarily correlated to SWC, evapotranspiration, gas exchange parameters, proline and root transverse diameter) could explain 11.8%, PC3 (primarily correlated to malondialdehyde, nitrate and vitamin C) could explain 9.1%, PC4 (primarily correlated to leaf length, leaf width, and root/shoot ratio) could explain 8.1%, PC5 (primarily correlated to reducing sugar) could explain 5.4%, and PC6 (primarily correlated to WUE_L_ and soluble protein) could explain 4.7%, with an 90.3% cumulative contribution rate (**Supplementary Tables 1, 2**). The CV, δ, SWC, and evapotranspiration (mainly composed of soil evaporation) were extrinsic environmental variables for cherry radish, and the results of the PCA indicated that the four environmental variables belonged to two categories of environmental variables, with CV and δ included in PC1, indicating soil water stability, and SWC and evapotranspiration contained in PC2, representing water content properties (because SWC was the basis of soil evaporation, with a high correlation). The FW conditions (CI1, CI2 and CI3) and SW conditions (NPI0, NPI1 and NPI2) were separated by PC1, where FW treatments were generally clustered more to the left and SW treatments more to the right on the plot. The preceding results indicated that soil water influenced the growth and development of cherry radish in two ways: soil water stability and SWC level.

### 3.9 Partial least squares path model between soil water and plant indices

**Figure 5:**
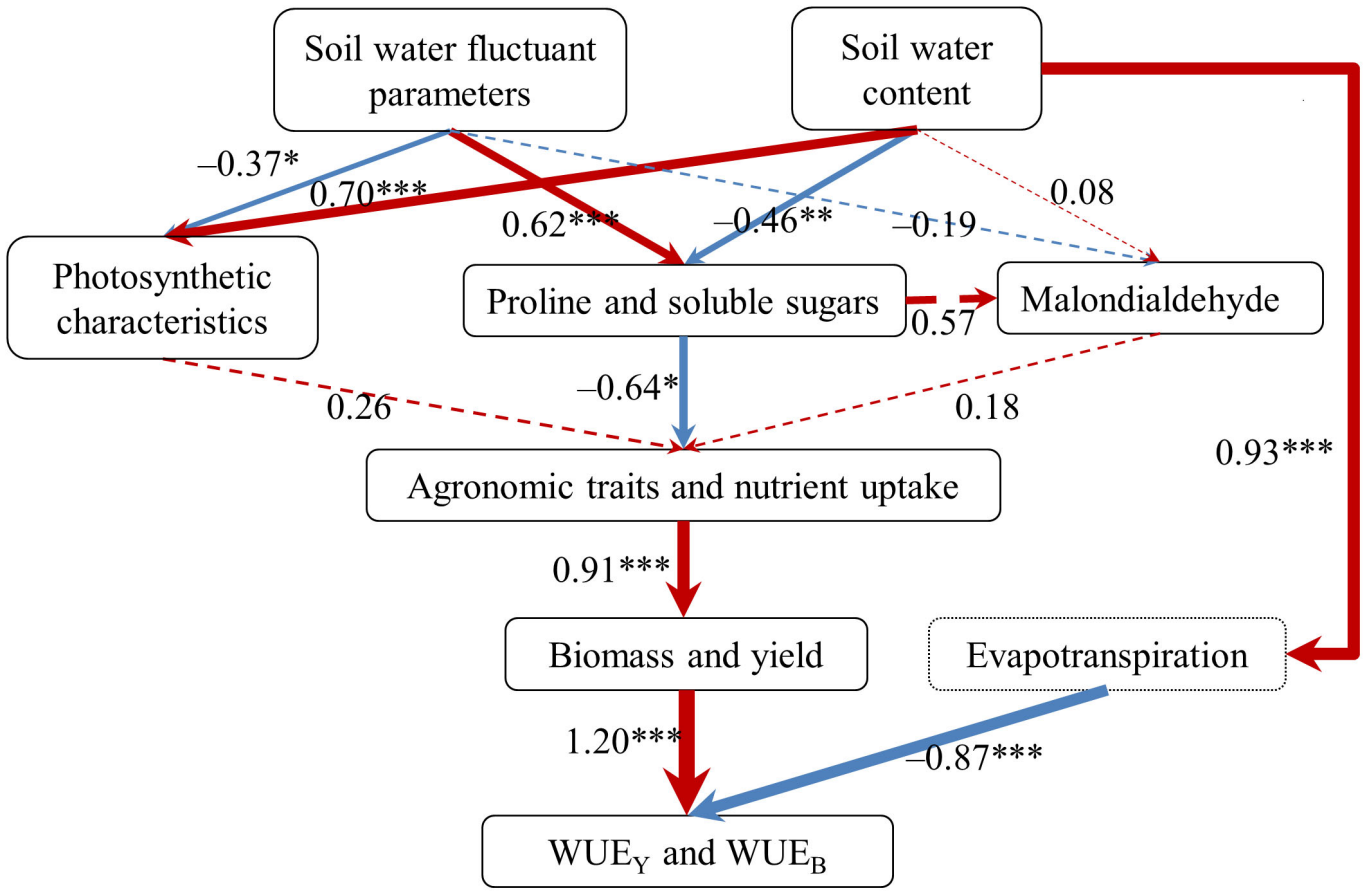
The partial least squares path analysis model (PLS-PM) was used to analyze how soil water affected the yield and water use efficiency of cherry radish. The path coefficients were represented by the width of the arrows. The red color indicated a positive effect, and the blue color indicated a negative effect. The solid arrows indicated significant effects (*P < 0.05; **P < 0.01; ***P < 0.001), and the dotted line indicated no significant effect (*P* > 0.05). WUE_Y_, yield water use efficiency; WUE_B_, biomass water use efficiency. The quality of the PLS-PM was evaluated by examining the goodness-of-fit index (GOF), and the GOF of this model was 0.79. The data used in the model were the data from the harvest periods.

Using PLS-PM analysis, the relationships between soil water fluctuant parameters, SWC, physiological characteristics, agronomic traits, biomass and yield, nutrient uptake, WUE_Y_ and WUE_B_ were integrated (**Figure 5**). Results revealed that soil water fluctuant parameters had significant adverse effects on the photosynthetic characteristics of cherry radish with a path coefficient of –0.37, whereas SWC had a significant positive effect with a path coefficient of 0.70. The soil water fluctuant parameters had significant positive effects on the water physiological indicators of cherry radish with a path coefficient of 0.62, which demonstrated that leaf water physiological indicators of cherry radish were highly influenced by soil water stability; whereas SWC had a significant adverse effect with a path coefficient of –0.46. The proline and soluble sugars had a significant adverse effect on agronomic traits and nutrient uptake with a path coefficient of –0.64, and the agronomic traits and nutrient uptake had a significant positive effect on the cherry radish biomass and yield with a path coefficient of 0.91. The biomass and yield had a significant positive effect on the WUE_Y_ and WUE_B_, with a path coefficient was 1.20; whereas evapotranspiration had a significant negative effect, with a path coefficient of –0.87. The PLS-PM exhibited that soil water stability and SWC had an impact on cherry radish’s proline and soluble sugars directly, as well as indirectly on its agronomic traits and nutrient uptake, which in turn influenced the biomass and yield of cherry radish, finally influenced the WUE_Y_ and WUE_B_. Particularly, soil water stability had an indirect effect on the agronomic traits and nutrient uptake of cherry radish by affecting its water physiological indicators, thereby affecting the final biomass and yield.

## 4 Discussion

### 4.1 Stable soil water enhances plant photosynthesis and avoids suffering physiological damage

Gas exchange is the foundation of plant biomass production, and the regulation of soil water on photosynthetic activity can greatly affect crop biomass and yield (Ali et al., 2019; Liu et al., 2019). According to Wang (2020), SW improved the photosynthesis of romaine lettuces and a similar phenomenon was observed in our findings. In addition, studies have found that under steady water supply, significant changes were observed in cell components linked to maize photosynthesis, including chloroplasts, plastids, and thylakoids, and genes such as *psbE*, *psbF*, *psbA*, and *psbD* related to photosystems were highly regulated (Zhang et al., 2021b). This may explain why SW increased the crop Photo. In contrast to Li et al. (2023), the results of this study demonstrated that SW improved the Photo of cherry radish compared to FW, but had no significant impact on WUE_L_. Besides, our findings demonstrated that photosynthetic parameters of cherry radish were highly influenced by soil water stability, and that SW was better suited to improving crop gas exchange parameters (**Figure 4A**), which facilitated the accumulation of photosynthetic products and increased yield.

Previous studies have shown that in respond to water stress, plants simultaneously accumulate more proline, malondialdehyde, and soluble sugars (Al-Yasi et al., 2020; Wahab et al., 2022). It has been reported that alternating soil water treatment was found to considerably increase the contents of proline, malondialdehyde and soluble sugars in maize roots when compared to the SW status under NPI treatments (Zhang et al., 2022a; Zhang et al., 2022b). Furthermore, studies have shown that stabilizing soil moisture alleviated maize’s short-term water stress (Niu et al., 2022). Importantly, proline and malondialdehyde contents in cherry radish in FW were higher than those in SW under similar SWC. It was speculated that the fluctuation of soil water caused some damage to cherry radish. Additionally, our results suggesting that the lower SWC and the stronger the soil moisture fluctuation, the more proline was accumulated in plants (**Table 3** and **Figure 4A**). However, our study found that the higher SWC and the stronger the soil water stability, the greater the soluble sugars content was accumulated in plants (**Figure 4A**), which was dissimilar to what was observed in the literature of Wahab et al. (2022). This could be as a result of a greater Cond being supported by a suitable and stable water supply, which raised the concentration of sugar that entered the phloem and increased the soluble sugars content (Gao et al., 2021). Besides, the PLS-PM exhibited that the proline and soluble sugars of cherry radish were significantly positively impacted by soil water fluctuant parameters (**Figure 5**), which demonstrated that soil water stability had a major impact on the leaf water physiological indicators of cherry radish. Prior research has found that, in response to variations in soil water, the proline content of maize roots and leaves did not differ significantly, whereas malondialdehyde content in maize roots was significantly greater than that in maize leaves, indicating that the relationship between plant physiological indicators and soil moisture status was related to crop organs (Niu et al., 2022). However, only the leaves physiological indices of cherry radish were investigated in this study; further research is needed to determine how the various organs of cherry radish respond to soil water stability.

### 4.2 Stable soil water motivates the assimilation of underground parts in root vegetables

The results of this study likely demonstrated that the root yield of cherry radishes decreased as SWC decreased, which was consistent with the findings of a previous study (Wu et al., 2021). Studies have demonstrated that under SW conditions, agronomic traits such as plant height, stem thickness, and leaf area of maize, romaine lettuces, flue-cured tobacco, and Chinese cabbage were enhanced, resulting in increased crop yield (Li et al., 2017; Wang, 2020; Yang et al., 2021; Yang et al., 2022b). Interestingly, studies have showed that SW inhibited the redundant growth of maize roots (Zhang et al., 2022b), and that SW could reduce root/shoot ratio of romaine lettuces in comparison to FW (Wang, 2020). Nevertheless, our findings indicated that SW and FW had similar effects on the root/shoot ratio of cherry radish, and did not significantly inhibit the growth and development of the radish underground portion, but increased root biomass (**Table 4**). The results were supported by plant growth metrics, which showed that SW resulted in greater overall plant growth in comparison to FW. By comparing the results of this study with those of previous research (Albacete et al., 2014; Stagnari et al., 2018; Zhang et al., 2022b), it was found that soil water stability regulated source-library relationships, and that SW promoted the distribution of assimilate to the economic organs of plants and improved crop yield than FW. Additionally, our study revealed that the more stable the soil water, the more conducive it was to the root crop growth and development (**Figure 4**).

### 4.3 Stable soil water promotes the nutrient uptake of root vegetables via producing biomass

Plant nutrient uptake, use, and transfer are impacted by soil water (Qu et al., 2020). Studies have shown that SW promoted the uptake and utilization of nitrogen, phosphorus and potassium by crown daisy and romaine lettuces (Wang, 2020; Yang et al., 2020). The results of this study proved that compared to FW, SW increased the nitrogen uptake of cherry radish by 23.6–60.9%, phosphorus uptake by 32.8–80.1%, and potassium uptake by 6.52–55.7%, which was consistent with the previous results (Yang et al., 2022b). Notably, our study found that the increase in nutrient uptake by plants was more attributable to the increase in biomass than the improvement in nutrient content (**Table 6**), indicating that SW promoted the radish nutrient uptake via producing biomass, which was supported by the **Figures 4**, **5**. In addition, the results showed that soil water stability had no significant effect on nutrient use efficiency of radish, but was affected by the level of SWC (**Supplementary Table 3**). When SWC was about 85% FC, SW increased the nutrient use efficiency by 3.80–14.7% compared with FW; whereas when SWC was close to 80% FC, SW enhanced the nitrogen use efficiency by 4.23% and the potassium use efficiency by 3.75%, but decreased the phosphorus use efficiency by 13.9%. Studies have revealed that phosphorus uptake was directly proportional to the volume of soil almost with the field water holding capacity and the length of time it remained moist (Simpson and Pinkerton, 1989). Moreover, studies have demonstrated that NPI treatments could transport water and nutrients directly to crop roots in line with plant requirements (Yang et al., 2022a), and that SW may promote nutrient distribution uniformity and reduce nutrient loss (Gao et al., 2021; Li et al., 2021b). Furthermore, the PLS-PM results revealed that soil water stability had an indirect effect on the agronomic traits and nutrient uptake of cherry radish by affecting its water physiological indicators, thereby affecting the final biomass and yield (**Figure 5**), which also confirmed our hypothesis.

### 4.4 Stable soil water improves the WUE of cherry radish plants by increasing biomass

Water use efficiency reflects the energy conversion efficiency in plant production, which is a crucial indicator for determining the relationship between crop yield and water usage (Sun et al., 2018). Studies have shown that NPI treatments reduced water consumption by 53.4–67.8% and enhanced the WUE by 12.7–124.7% of *Capsicum annuum* L. when compared to watering treatments (Li et al., 2017). In our study, compared to CI treatments, NPI treatments increased the WUE_Y_ by 45.9–74.2% and WUE_B_ by 46.7–53.8%, respectively, in cherry radishes grown under similar SWC conditions. However, the WUE_Y_ and WUE_B_ calculations were based on evapotranspiration (Sun et al., 2018; Wang, 2020; Liao et al., 2022), which actually referred to the WUE in the field of irrigation technology. Since the soil evaporation component of evapotranspiration was not directly related to plant growth and development, WUE_Y_ and WUE_B_ are unable to fully reflect the effect of soil water stability on plant WUE. Nevertheless, the measured data in our study revealed that SW increased radish yield by 34.6–94.1%, biomass by 42.2–63.5%, and leaf Trmmol by 11.3–31.9%, when compared to FW. Importantly, the rate of increase in biomass and yield exceeded that of evapotranspiration, and this was further supported by the PLS-PM results (**Figure 5**), indicating that soil water stability increased cherry radish WUE by promoting its biomass and yield. Moreover, the results confirmed that soil water fluctuant parameters had a negative relationship with the WUE_Y_ and WUE_B_ of cherry radish (**Figure 4**). This also demonstrated that SW could improve soil water conditions to allow cherry radish to use water more effectively. It is necessary to use transcriptome or metabolomics to further study the molecular mechanism of increasing WUE in root vegetables by stable water supply mode.

## 5 Conclusion

In this study, NPI and CI were used to create stable and fluctuating soil water, respectively, while maintaining similar average SWC conditions between them. A pot experiment was conducted to examine the agronomic traits, gas exchange, proline, malondialdehyde, soluble sugars, biomass, yield, quality, nutrient uptake and water use of cherry radish under different soil water states.

(i) Under similar SWC conditions, as compared to FW, SW improved the Photo, Cond and Trmmol of cherry radish, decreased the proline and malondialdehyde contents of the leaves, and increased the soluble sugars content, resulting in improved growth and development of cherry radish.
(ii) Under similar SWC conditions, SW was more conducive to the accumulation of cherry radish biomass and nutrient uptake than FW. Stable soil water significantly increased the radish yield by 35–94%, with a similar effect as FW on radish quality and root/shoot ratio.
(iii) Soil water stability affected directly the root vegetable water physiological indicators, and indirectly its agronomic traits and nutrient uptake, which in turn influenced the crop biomass and yield, as well as WUE.

When SWC was comparable, SW was more conducive to the growth and development of crops than FW. Stable soil water could promote the distribution of assimilate to the economic parts of plants and increase crop yield, resulting in a great potential for enhancing WUE. This study fills the gap where soil water stability affects root crop growth, and provides a new perspective for improving agronomy of root crops and WUE through managing soil water stability.

## Data availability statement

The original contributions presented in the study are included in the article/**Supplementary Material**. Further inquiries can be directed to the corresponding author.

## Author contributions

GL: Conceptualization, Data curation, Writing – original draft, Writing – review & editing. GZ: Data curation, Formal analysis, Investigation, Writing – review & editing. JL: Supervision, Writing – review & editing. ZW: Methodology, Writing – review & editing. HL: Conceptualization, Supervision, Writing – review & editing. RZ: Supervision, Writing – review & editing. KY: Project administration, Writing – review & editing.
