# Supplementary material for: Effects of stable and fluctuating soil water on the agronomic and biological performance of root vegetables
Source: Front Plant Sci. 2024 Feb 14;15:1325078. doi: 10.3389/fpls.2024.1325078 (PMC10899879; doi:10.3389/fpls.2024.1325078)
Supplement: Supplementary file 1 [file Table_1.docx]

Supplementary Material

# Supplementary Tables

**Supplementary Table 1**

Eigenvalues and variances of principal component analysis.

| Principal Component Number | Eigenvalue | Percentage of Variance (%) | Cumulative (%) |
| --- | --- | --- | --- |
| PC1 | 14.37 | 51.33 | 51.33 |
| PC2 | 3.29 | 11.76 | 63.09 |
| PC3 | 2.54 | 9.06 | 72.16 |
| PC4 | 2.26 | 8.06 | 80.21 |
| PC5 | 1.50 | 5.36 | 85.57 |
| PC6 | 1.32 | 4.70 | 90.27 |

Note: PCn indicated the extracted principal component.

**Supplementary Table 2**

Principal component loading coefficients of principal component analysis.

| Item | Loading coefficients | | | | | | Commonalities |
| --- | --- | --- | --- | --- | --- | --- | --- |
|  | PC1 | PC2 | PC3 | PC4 | PC5 | PC6 |  |
| SWC | 0.12 | 0.95 | 0.13 | 0.15 | –0.04 | 0.08 | 0.97 |
| CV | –0.89 | –0.23 | 0.17 | –0.08 | –0.20 | –0.02 | 0.92 |
| δ | –0.87 | –0.17 | 0.09 | –0.07 | –0.14 | –0.21 | 0.87 |
| Height | 0.62 | 0.41 | –0.03 | 0.56 | 0.21 | 0.06 | 0.92 |
| LL | 0.54 | 0.19 | 0.13 | 0.62 | 0.46 | –0.10 | 0.95 |
| LW | 0.54 | 0.17 | 0.14 | 0.61 | 0.49 | –0.16 | 0.98 |
| Photo | 0.40 | 0.79 | –0.23 | 0.01 | 0.30 | 0.13 | 0.95 |
| Cond | 0.36 | 0.84 | –0.07 | 0.03 | 0.29 | –0.02 | 0.93 |
| Trmmol | 0.25 | 0.81 | –0.17 | –0.16 | 0.30 | –0.24 | 0.93 |
| WUE_L_ | 0.32 | 0.02 | –0.26 | 0.31 | 0.10 | 0.80 | 0.92 |
| Proline | –0.36 | –0.55 | 0.24 | –0.06 | –0.48 | 0.17 | 0.74 |
| MDA | 0.10 | –0.34 | 0.73 | –0.17 | –0.36 | –0.11 | 0.82 |
| SS | 0.62 | 0.49 | 0.21 | 0.10 | –0.14 | 0.23 | 0.75 |
| Nitrate | –0.10 | 0.03 | 0.89 | –0.14 | –0.05 | –0.06 | 0.83 |
| VC | –0.23 | 0.06 | 0.71 | 0.22 | 0.06 | 0.13 | 0.63 |
| RS | –0.18 | –0.15 | 0.10 | –0.02 | –0.89 | –0.23 | 0.90 |
| SP | –0.27 | 0.01 | 0.50 | –0.11 | 0.13 | 0.69 | 0.83 |
| TN | 0.78 | 0.50 | –0.03 | 0.28 | 0.01 | 0.00 | 0.93 |
| TP | 0.88 | 0.40 | –0.11 | 0.14 | 0.10 | –0.04 | 0.98 |
| TK | 0.84 | 0.44 | –0.06 | 0.14 | 0.06 | –0.07 | 0.93 |
| Yield | 0.70 | 0.62 | –0.09 | –0.10 | 0.24 | –0.12 | 0.96 |
| RD | 0.60 | 0.61 | –0.02 | –0.13 | 0.37 | –0.22 | 0.94 |
| RL | 0.73 | 0.61 | –0.01 | 0.04 | –0.02 | 0.09 | 0.91 |
| Biomass | 0.81 | 0.52 | –0.10 | 0.16 | 0.02 | 0.05 | 0.97 |
| R/S | 0.05 | 0.01 | 0.14 | –0.92 | 0.14 | –0.21 | 0.93 |
| ET | 0.06 | 0.93 | 0.08 | 0.21 | –0.08 | 0.08 | 0.94 |
| WUE_Y_ | 0.87 | 0.03 | –0.18 | –0.20 | 0.34 | –0.21 | 0.98 |
| WUE_B_ | 0.91 | –0.31 | –0.18 | 0.05 | 0.08 | 0.00 | 0.97 |

Note: PCn indicated the extracted principal component. SWC, soil water content; CV, variation coefficient; δ, fluctuation coefficient; Height, plant height; LL, leaf length; LW, leaf width; Photo, photosynthetic rate; Cond, stomatal conductance; Trmmol, transpiration rate; WUE_L_, leaf water use efficiency; Proline, free proline content; MDA, malondialdehyde content; SS, soluble sugars content; Nitrate, nitrate content; VC, vitamin C content; RS, reducing sugars content; SP, soluble protein content; TN, total nitrogen uptake; TP, total phosphorus uptake; TK, total potassium uptake; Yield, fruit yield; RD, root transverse diameter; RL, root longitudinal diameter; Biomass, total biomass; R/S, root/shoot ratio; ET, evapotranspiration; WUE_Y_, yield water use efficiency; WUE_B_, biomass water use efficiency.

**Supplementary Table 3**

Nutrient use efficiency of cherry radish under different treatments.

| Treatment | Nutrient use efficiency (g g^–1^) | | |
| --- | --- | --- | --- |
|  | N | P | K |
| NPI0 | 10.7 ± 1.52ab | 158 ± 8.31ab | 14.1 ± 1.47ab |
| NPI1 | 12.5 ± 1.49a | 166 ± 15.3a | 15.0 ± 0.45a |
| NPI2 | 10.6 ± 1.19ab | 138 ± 16.5b | 13.2 ± 1.55ab |
| CI1 | 10.9 ± 1.23ab | 160 ± 9.24ab | 13.1 ± 1.42ab |
| CI2 | 10.1 ± 0.26b | 160 ± 6.28ab | 12.7 ± 0.53b |
| CI3 | 9.50 ± 0.42b | 159 ± 15.5ab | 12.2 ± 0.80b |

Note: NPI, negative pressure irrigation; CI, conventional irrigation; N, nitrogen; P, phosphorus; K, potassium. Different lowercase letters in the same column indicate significant differences among treatments at the *P* < 0.05 level.
